# The influence of femoral tunnel length on graft rupture after anterior cruciate ligament reconstruction

**DOI:** 10.1007/s10195-017-0448-9

**Published:** 2017-02-18

**Authors:** Luiz Gabriel Betoni Guglielmetti, Leandro Girardi Shimba, Leonardo Cantarelli do Santos, Fabrício Roberto Severino, Nilson Roberto Severino, Patrícia Maria de Moraes Barros Fucs, Ricardo de Paula Leite Cury

**Affiliations:** 1 Knee Group of the Department of Orthopedics and Traumatology of Santa Casa de Misericórdia of São Paulo, Santa Casa School of Medical Sciences, São Paulo–Fernandinho Simonsen Pavilion, São Paulo, Brazil; 2 Pascal, 1292, Ap91, Campo Belo, São Paulo, São Paulo, 04616-004 Brazil

**Keywords:** ACL reconstruction, Hamstring tendon, Graft failure, Tunnel length

## Abstract

**Background:**

For ACL reconstruction, the minimum length of the femoral tunnel and the flexor tendon graft length needed within the tunnel for proper integration have not been defined. The aim of this study was to assess whether a short tunnel is a risk factor for poor prognosis and re-rupture by comparing the outcomes of patients with short femoral tunnels to those of patients with longer tunnels.

**Materials and methods:**

A retrospective observational study of 80 patients who underwent ACL reconstruction using flexor tendons via the medial transportal or transtibial technique was performed. Patients were categorized according to the amount of graft within the tunnel: ≤1.5 versus >1.5 cm; ≤2 versus >2 cm; ≤2.5 versus >2.5 cm; and ≤1.5 versus >2.5 cm. Patients were evaluated 2 years after surgery by performing a physical examination (Lachman, pivot shift and anterior drawer tests), using a KT1000 arthrometer, calculating objective and subjective International Knee Documentation Committee scores, conducting the Lysholm score, and recording re-ruptures.

**Results:**

Of the 80 operated patients, nine were lost to follow-up. Comparative assessment of the patients with different amounts of graft within the tunnel indicated no significant differences in the evaluated outcomes, except for positive Lachman test results, which were more frequent in patients with tunnels with ≤2 cm of graft than in those with tunnels with >2 cm of graft.

**Conclusion:**

The amount of graft within the femoral tunnel does not appear to be a risk factor for clinical instability of the knee or re-rupture of the graft. Level of evidence: case series, level IV.

**Level of evidence:**

Case series, level IV.

## Introduction

Successful reconstruction of the anterior cruciate ligament (ACL) is related to several factors, such as the correct positioning of the tunnels, treatment of associated lesions, fixation method, graft quality, and proper rehabilitation [1, 2]. Anatomical reconstruction has produced greater rotational and anterior control than the transtibial technique [3–5], and the technique performed through the medial portal is one option [6, 7]. Despite its advantages, several problems are associated with the transportal technique, such as chondral injury of the medial femoral condyle and the presence of a short femoral tunnel [6, 8–10].

Fixation of the flexor tendon graft to bone is an important factor related to the success of ACL reconstruction surgery. However, the process of bone graft incorporation remains unclear [11, 12]. A number of histological studies have suggested that the continuity of collagen fibers between the graft and the bone is progressively reestablished [11, 13–18]. However, neither the minimum length of the femoral tunnel in ACL reconstruction nor the amount of flexor tendon graft needed within the tunnel for proper integration has been defined [20]. In addition, studies on this subject have been performed in animal models [11, 19–21].

The objective of this study was to retrospectively compare the incidence of new ruptures and the clinical outcomes (objective and subjective) of surgical patients with a short graft length within the femoral tunnel to those of patients with a longer graft length within this tunnel.

## Materials and methods

In this observational study, we retrospectively evaluated a cohort of 80 patients who underwent ACL reconstruction between 2010 and 2012 at a single center. The patients were operated on by the same surgeon and were followed as outpatients. Informed consent was obtained from each participant included in the study. The inclusion criteria were unilateral ACL injury; closed physis; age <40 years; no previous surgery on the affected knee (except arthroscopic meniscectomy); no severe degenerative changes on arthroscopy; <1 year since injury; and no morbid obesity. These 80 patients were selected for another study, a randomized controlled trial comparing two different techniques: transportal and transtibial ACL reconstruction. That study was submitted to another journal, and we are waiting for it to be accepted. Because of the technique (transportal) and fixation device used, we had some patients with a short graft length within the femoral tunnel, which motivated us to study how much graft is needed inside the tunnel, and we did not find an answer in the literature. To answer this question, we decided to retrospectively evaluate these patients in terms of clinical results and re-rupture.

Eighty patients (59 men and 21 women), corresponding to 43 right knees and 37 left knees, underwent surgery. The mean age of the patients was 24 years, ranging from 15 to 40 years. The average time from injury to surgery was 6.5 months (minimum 3 weeks, maximum 1 year). The mean preoperative subjective International Knee Documentation Committee (IKDC) score was 66.74 (minimum 37 and maximum 90), and the mean average preoperative Lysholm score was 69.25 (minimum 36 and maximum 89).

Clinical evaluations were performed before surgery and at 1, 2, 4, 6, 12, and 24 months after surgery. The objective evaluation was performed using a KT1000™ arthrometer (MEDmetric, San Diego, CA, USA) at 20° of flexion with a 133-N load; additionally, the Lachman test, the anterior drawer test, and the pivot shift test were performed, and the objective IKDC score was calculated [22]. The subjective evaluation consisted of calculating the subjective IKDC and Lysholm scores [23]. Re-ruptures were defined as new knee sprains associated with clinical instability.

### Surgical technique

**Fig. 1:**
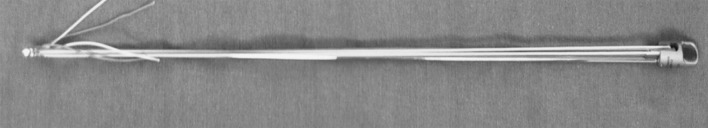
Endo Tunnel Device (ETD^®^)

**Fig. 2:**
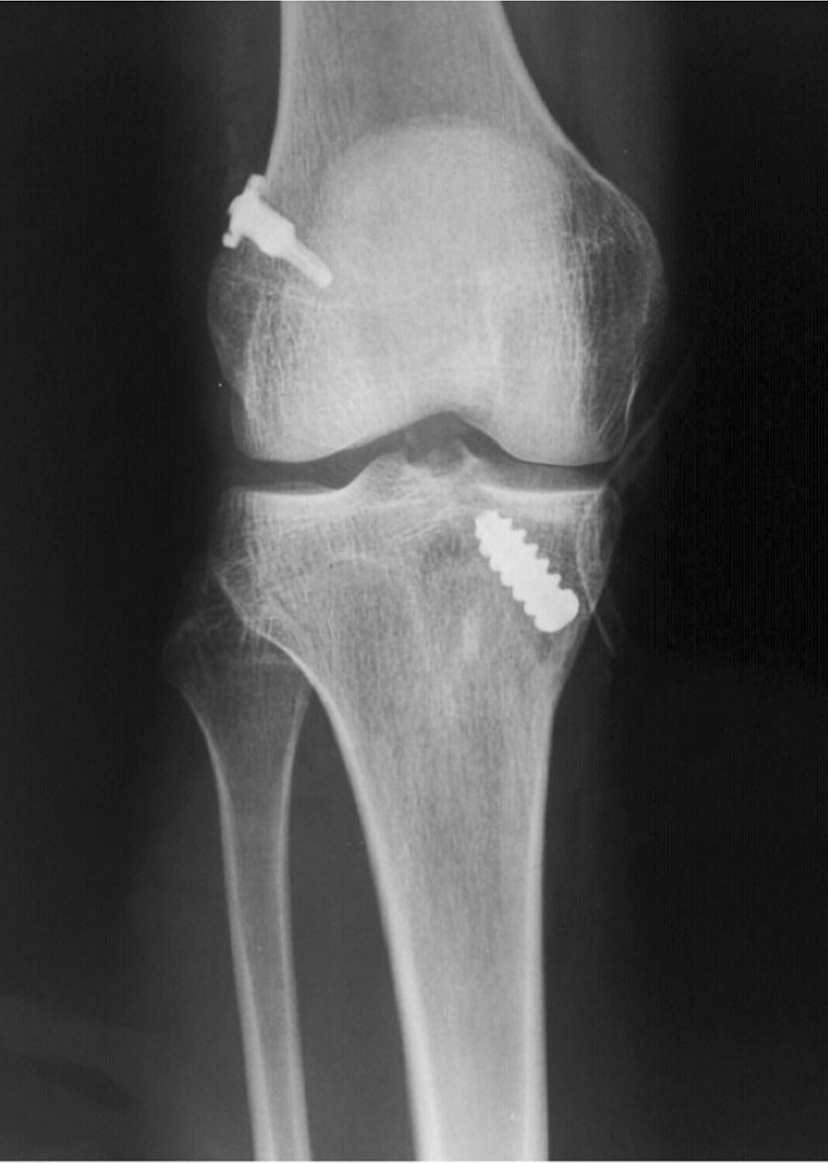
Postoperative anteroposterior radiograph of the right knee subjected to ACL reconstruction, showing femoral fixation with the ETD^®^

**Fig. 3:**
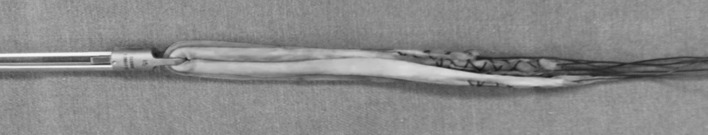
ETD^®^ with the graft (semitendinosus and gracilis tendons) prepared

Arthroscopy was performed, followed by treatment of possible meniscal and chondral injuries and ACL reconstruction via flexor tendon graft fixation to the tibia using a metallic interference screw. In the femur, the Endo Tunnel Device^®^ (ETD) (Proind, Cotia, São Paulo, Brazil)—a suspension device for femoral fixation—was used (Figs. 1, 2, 3). The ETD has various implant diameters and lengths. The diameter varies from 7 to 9 mm, and the length can be 20, 25, 30, or 35 mm. The parameter used to select the length of the button was the femoral length. The goal was to place 25 mm of graft within the tunnel; therefore, during the surgery, the button length was calculated by subtracting 25 mm from the femoral tunnel length.

Forty patients were operated on using the transtibial technique and 40 using the transportal technique.

### Rehabilitation

All patients received the same rehabilitation protocol, as determined by the knee group associated with the physiotherapy group of our institution.

Given that the minimum length of the ETD^®^ is 2 cm, there were cases with short tunnels in which little graft remained in the femoral tunnel. The length of the femoral tunnel was measured in all cases using a special ruler during the surgery, and the remaining amount of graft within the tunnel was calculated by subtracting the length of the ETD^®^. The patients were categorized according to this measure as follows in order to compare clinical outcomes and the incidence of re-ruptures: patients with ≤1.5 cm of graft within the tunnel versus patients with >1.5 cm; ≤2 versus >2 cm; ≤2.5 versus >2.5 cm; and ≤1.5 versus >2.5 cm.

### Statistics

Data were statistically analyzed using SPSS version 13.0 for Windows. For descriptive statistical analysis, qualitative variables are expressed as frequencies (number and percentage) and visually. The quantitative variables are expressed using summary measures (mean, median, standard deviation, minimum and maximum). For comparisons of two qualitative variables, the chi-squared or Fisher’s exact test was used. For comparisons of one qualitative variable and quantitative variable, Student’s *t* test (parametric) or the Mann–Whitney test (nonparametric) was used at the 5% significance level.

## Results

Of the 80 patients, 71 were re-evaluated 2 years after surgery; the 9 patients who did not respond were considered lost to follow-up. Such losses occurred for three reasons: death (1 patient), incorrect contact information (3 patients), and non-attendance of the re-examination (5 patients). Therefore, a total of 71 patients with 2 years of follow-up were assessed. Of these, 37 underwent surgery using the transportal technique and 34 using the transtibial technique.

The mean length of the femoral tunnel was 4.98 cm in the transtibial group (minimum 4 cm and maximum 6.5 cm) and 3.99 cm in the transportal group (minimum 2.9 cm and maximum 5 cm) (*p* < 0.001; Student’s *t* test). The mean length of the graft within the femoral tunnel was 2.91 cm in the transtibial group (minimum 2.2 cm and maximum 4 cm) and 2.27 cm in the transportal group (minimum 0.9 cm and maximum 2.7 cm) (*p* < 0.001; Student’s *t* test).

The following groups were compared regarding the incidence of re-rupture, the results of a physical examination (Lachman, pivot shift, and anterior drawer tests), using a KT1000 arthrometer, the objective and subjective IKDC scores, and the Lysholm scores result: patients with ≤1.5 cm of graft within the femoral tunnel versus >1.5 cm; ≤2 versus >2 cm; ≤2.5 versus >2.5 cm; and ≤1.5 versus >2.5 cm. Because two techniques were used (transtibial and transportal) and because the transportal technique created shorter tunnels, the comparative evaluations mentioned above were performed on the transportal and transtibial subgroups together and on only the transportal subgroup. Tables 1, 2, 3, and 4 show the results and statistical tests used to analyze the 71 patients. Tables 5, 6, 7, and 8 show the same evaluation for the 37 patients in the transportal subgroup. To evaluate the results of the physical examination (Lachman, pivot shift, and anterior drawer tests), due to the number of variables (0+, 1+, 2+, 3+) and to facilitate statistical analysis, the physical evaluation results were divided into normal (0+) and abnormal (1+, 2+, or 3+). The objective IKDC scores were divided into group 1 (IKDC A) and group 2 (IKDC B, C, or D). No significant difference was observed in most outcomes (re-rupture, KT1000 arthrometry, physical examination, objective and subjective IKDC scores, and Lysholm scores results). The exception was the Lachman test, in which the comparison of patients with ≤2 cm of graft within the femoral tunnel to those with >2 cm revealed more cases with a positive Lachman test result in the subgroup with a smaller graft length within the femoral tunnel (*p* = 0.025, Fisherʼs exact test).

**Table 1:** Comparison of all 71 patients for re-rupture, Lachman test, pivot shift test, anterior drawer test, KT1000 test, objective IKDC, subjective IKDC, Lysholm score: patients with ≤1.5 cm of graft within the femoral tunnel versus >1.5 cm

	≤1.5 cm	>1.5 cm	*p*
Re-rupture	1 (4)	5 (67)	0.303 (Fisher’s test)
Lachman test			
Normal	2	52	–
Abnormal	2	15	–
Total	4	67	0.241 (Fisher’s test)
Pivot shift test			
Normal	2	54	–
Abnormal	2	13	–
Total	4	67	0.194 (Fisher’s test)
Anterior drawer test			
Normal	2	48	–
Abnormal	2	21	–
Total	4	67	0.576 (Fisher’s test)
KT-1000 test	1.63	1.31	0.808 (Mann–Whitney test)
Objective IKDC			
A	2	45	–
B, C, e D	2	22	–
Total	4	67	0.599 (Fisher’s test)
Subjective IKDC	93.25	91.6	0.547 (Mann–Whitney test)
Lysholm score	92.5	92.07	0.661 (Mann–Whitney test)

**Table 2:** Comparison of all 71 patients for re-rupture, Lachman test, pivot shift test, anterior drawer test, KT1000 test, objective IKDC, subjective IKDC, Lysholm score: patients with ≤2.0 cm of graft within the femoral tunnel versus >2.0 cm

	≤2.0 cm	>2.0 cm	*p*
Re-rupture	2 (13)	4 (58)	0.301 (Fisher’s test)
Lachman test			
Normal	8	46	–
Abnormal	5	12	–
Total	13	58	0.278 (Fisher’s test)
Pivot shift test			
Normal	9	47	–
Abnormal	4	11	–
Total	13	58	0.452 (Fisher’s test)
Anterior drawer test			
Normal	8	42	–
Abnormal	5	16	–
Total	13	58	0.507 (Fisher’s test)
KT-1000 test	1.42	1.31	0.945 (Mann–Whitney test)
Objective IKDC			
A	8	39	–
B, C, e D	5	19	–
Total	13	58	0.751 (Fisher’s test)
Subjective IKDC	91.77	91.67	0.681 (Mann–Whitney test)
Lysholm score	92.31	92.05	0.531 (Mann–Whitney test)

**Table 3:** Comparison of all 71 patients for re-rupture, Lachman test, pivot shift test, anterior drawer test, KT1000 test, objective IKDC, subjective IKDC, Lysholm score: patients with ≤2.5 cm of graft within the femoral tunnel versus >2.5 cm

	≤2.5 cm	>2.5 cm	*p*
Re-rupture	3 (40)	3 (31)	1 (Fisher’s test)
Lachman test			
Normal	30	24	–
Abnormal	10	7	–
Total	40	31	1 (Chi-squared test)
Pivot shift test			
Normal	31	25	–
Abnormal	9	6	–
Total	40	31	0.747 (Fisher’s test)
Anterior drawer test			
Normal	27	23	–
Abnormal	13	8	–
Total	40	31	0.540 (Fisher’s test)
KT-1000 test	1.19	1.52	0.353 (Student’s *t* test)
Objective IKDC			
A	24	23	–
B, C, e D	16	8	–
Total	40	31	0.210 (Chi-squared test)
Subjective IKDC	93	90	0.223 (Student’s *t* test)
Lysholm score	93.38	90.45	0.193 (Student’s *t* test)

**Table 4:** Comparison of all 71 patients for re-rupture, Lachman test, pivot shift test, anterior drawer test, KT1000 test, objective IKDC, subjective IKDC, Lysholm score: patients with ≤1.5 cm of graft within the femoral tunnel versus >2.5 cm

	≤1.5 cm	>2.5 cm	*p*
Re-rupture	1 (4)	3 (31)	0.399 (Fisher’s test)
Lachman test			
Normal	2	24	–
Abnormal	2	7	–
Total	4	31	0.268 (Fisher’s test)
Pivot shift test			
Normal	2	25	–
Abnormal	2	6	–
Total	4	31	0.218 (Fisher’s test)
Anterior drawer test			
Normal	2	23	–
Abnormal	2	8	–
Total	4	31	0.561 (Fisher’s test)
KT-1000 test	1.63	1.31	0.958 (Mann–Whitney test)
Objective IKDC			
A	2	23	–
B, C, e D	2	8	–
Total	4	31	0.561 (Fisher’s test)
Subjective IKDC	93.25	90	0.480 (Mann–Whitney test)
Lysholm score	92.5	90.45	0.567 (Mann–Whitney test)

**Table 5:** Comparison of only transportal patients (35), for re-rupture, Lachman test, pivot shift test, anterior drawer test, KT1000 test, objective IKDC, subjective IKDC, Lysholm score: patients with ≤1.5 cm of graft within the femoral tunnel versus >1.5 cm

	≤1.5 cm	>1.5 cm	*p*
Re-rupture	1 (4)	2 (33)	0.298 (Fisher’s test)
Lachman test			
Normal	2	28	–
Abnormal	2	5	–
Total	4	33	0.155 (Fisher’s test)
Pivot shift			
Normal	2	28	–
Abnormal	2	5	–
Total	4	33	0.155 (Fisher’s test)
Anterior drawer test			
Normal	2	24	–
Abnormal	2	9	–
Total	4	33	0.567 (Fisher’s test)
KT-1000 test	1.63	1.18	0.725 (Mann–Whitney test)
Objective IKDC			
A	2	25	–
B, C, e D	2	8	–
Total	4	33	0.291 (Fisher’s test)
Subjective IKDC	93.25	92.58	0.588 (Mann–Whitney test)
Lysholm score	92.5	92.85	0.659 (Mann–Whitney test)

**Table 6:** Comparison of only transportal patients (35), for re-rupture, Lachman test, pivot shift test, anterior drawer test, KT1000 test, objective IKDC, subjective IKDC, Lysholm score: patients with ≤2.0 cm of graft within the femoral tunnel versus >2.0 cm

	≤2.00 cm	>2.00 cm	*p*
Re-rupture	2 (12)	1 (25)	0.241 (Fisher’s test)
Lachman test			
Normal	7	23	–
Abnormal	5	2	–
Total	12	25	0.025 (Fisher’s test)
Pivot shift			
Normal	8	22	–
Abnormal	4	3	–
Total	12	25	0.183
Anterior drawer test			
Normal	7	19	–
Abnormal	5	6	–
Total	12	25	0.443
KT-1000 test	1.63	1.04	0.808 (Mann–Whitney test)
Objective IKDC			
A	7	20	–
B, C, e D	5	5	–
Total	12	25	0.240 (Fisher’s test)
Subjective IKDC	91.33	93.28	0.405 (Mann–Whitney test)
Lysholm score	91.83	93.28	0.757 (Mann–Whitney test)

**Table 7:** Comparison of only transportal patients (35), for re-rupture, Lachman test, pivot shift test, anterior drawer test, KT1000 test, objective IKDC, subjective IKDC, Lysholm score: patients with ≤2.5 cm of graft within the femoral tunnel versus >2.5 cm

	≤2.5 cm	>2.5 cm	*p*
Re-rupture	3 (29)	0 (8)	1 (Fisher’s test)
Lachman test			
Normal	22	8	–
Abnormal	7	0	–
Total	29	8	0.308 (Fisher’s test)
Pivot shift			
Normal	22	8	–
Abnormal	7	0	–
Total	29	8	0.308 (Fisher’s test)
Anterior drawer test			
Normal	19	7	–
Abnormal	10	1	–
Total	29	8	0.391
KT-1000 test	1.22	1.25	0.791 (Mann–Whitney test)
Objective IKDC			
A	20	7	–
B, C, e D	9	1	–
Total	29	8	0.404
Subjective IKDC	92.38	93.63	0.373 (Mann–Whitney test)
Lysholm score	92.55	93.75	0.345 (Mann–Whitney test)

**Table 8:** Comparison of only transportal patients (35), for re-rupture, Lachman test, pivot shift test, anterior drawer test, KT1000 test, objective IKDC, subjective IKDC, Lysholm score: patients with ≤1.5 cm of graft within the femoral tunnel versus >2.5 cm

	≤1.5 cm	>2.5 cm	*p*
Re-rupture	1 (4)	0 (8)	0.333 (Fisher’s test)
Lachman test			
Normal	2	8	–
Abnormal	2	0	–
Total	4	8	0.091
Pivot shift			
Normal	2	8	–
Abnormal	2	0	–
Total	4	8	0.091
Anterior drawer test			
Normal	2	7	–
Abnormal	2	1	–
Total	4	8	0.236
KT-1000 test	1.63	1.25	0.791 (Mann–Whitney test)
Objective IKDC			
A	2	7	–
B, C, e D	2	1	–
Total	4	8	0.236
Subjective IKDC	93.25	91.6	0.864 (Mann–Whitney test)
Lysholm score	92.5	93.63	1 (Mann–Whitney test))

Re-ruptures occurred in 6 of the 71 patients evaluated (8%). Of these, re-ruptures occurred in 3 patients who received the transportal technique and 3 patients who received the transtibial technique. The amount of graft within the tunnel was 1.0, 2.0, and 2.5 cm in the 3 patients who received the transportal technique and 3.0 cm in all 3 patients who received the transtibial technique. Three re-ruptures occurred during physiotherapy (within the first 6 months of follow-up), two between 6 and 12 months of follow-up, and one at 16 months of follow-up.

Regarding surgical complications, there were 2 cases of superficial infection (transtibial subgroup) treated only with antibiotic therapy, which ultimately healed, and 1 case of arthrofibrosis (transportal subgroup) that required arthroscopy and manipulation under anesthesia, in which complete mobility was ultimately attained.

## Discussion

Reconstruction of the ACL using the medial transportal technique creates a shorter femoral tunnel than if the transtibial technique is used [4–6]. This finding, associated with the implant (ETD^®^) [24] used in this study, which has a minimum length of 2 cm, resulted in a limited graft length within the femoral tunnel. The amount of graft within the tunnel was arbitrarily divided into groups for comparison (≤1.5, ≤2, or ≤2.5 cm) because the minimum graft length necessary for proper integration is not specified in the literature [16]. Conducting a randomized controlled prospective study comparing different tunnel lengths and amounts of graft within the tunnel is not feasible for ethical reasons. Thus, an alternative approach is a retrospective analysis of cases that had a tunnel with a short graft length for some reason.

When all 71 patients were evaluated regarding the outcomes of physical examinations (Lachman, pivot shift, and anterior drawer tests), KT1000 assessments, objective and subjective IKDC score calculations, the Lysholm scores, and the incidence of re-ruptures, no significant differences were found between the groups. The transportal technique creates a different position and obliquity of the femoral tunnel compared to the transtibial technique, and it can affect the clinical outcome [5]. Because two different techniques were used in these patients (transtibial and transportal), possibly resulting in confounding differences between these subgroups, the transportal subgroup was also evaluated alone. However, no variations according to the graft length remaining were observed in this subgroup. The transportal subgroup contained only 37 patients, and this is an important study limitation because it represents a small sample size. This limitation generated the possibility of a type 2 statistical error (failure to detect a difference between groups when a difference exists).

This study included few cases with little graft within the femoral tunnel, but only one previous study in humans assessed the influence of hamstring autograft length in the femoral tunnel on outcomes following primary ACL reconstruction [25]. In that study, which retrospectively compared patients with at least 25 mm in the tunnel to patients with less than 25 mm in the tunnel, no clinical differences were found at two years after surgery. Yamazaki et al. [11] compared tibial tunnels with grafts measuring 5 mm to those with grafts of 15 mm in length in dogs and did not find any differences in ultimate failure load or linear stiffness of the graft 6 weeks after surgery. Zantop et al. [19] compared femoral tunnels with grafts measuring 15 to those with grafts of 25 mm in goats. Twelve weeks after the procedure, no difference in graft stiffness, ultimate failure load, or ultimate stress was found between the two groups. Yuan et al. [21] compared various situations regarding the amount of graft within the tunnels, evaluating reconstructions performed on dogs with 5, 9, 13, 17, 21, and 25 mm of graft within the tunnel. At 45, 90, and 180 days, the tunnels with 17 mm or more of graft exhibited better results in terms of maximum tensile strength and graft stiffness than the tunnels with 5, 9, or 13 mm of graft. In that study, 17 mm was considered the ideal graft length within the tunnel for ACL reconstruction. Thus, there are experimental models of this topic in the literature, but those models had many limitations because they used artificial situations and examined animals with bones and tendons with dimensions and structures that are distinct from those of human bones.

Considering the limitations of this study, these results must be interpreted with caution. Because this is a retrospective cohort study that aimed to identify risk factors, not cause–effect relationships, concluding that short tunnels do not cause joint instability and re-rupture is risky. In addition, it is impossible to define the minimum amount of graft needed within the femoral tunnel based on the results of this study mainly because the sample size is small, weakening the power of the statistical tests applied. We believe that these results raise the possibility that a minimum amount of 2–3 cm of graft within the femoral tunnel is an overestimate, and that a shorter graft length is sufficient for proper tunnel graft integration. Larger case series are necessary to support such a conclusion.

### Limitations

Short femoral tunnels were created only in the transportal group. All patients were evaluated by the same doctor who performed the surgery, who was aware of the technique used and the amount of graft within each patient. A total of 9 patients (11%) were lost to follow-up. Few patients had a short graft length within the tunnel, complicating the statistical analysis.
